# Increased cerebral cortex activation in stroke patients during electrical stimulation of cerebellar fastigial nucleus with functional near-infrared spectroscopy

**DOI:** 10.3389/fnins.2022.895237

**Published:** 2022-08-18

**Authors:** Haiyun Ma, Yujia Zhai, Zhen Xu, Shengnuo Fan, Xian Wu, Jing Xu, Shaoling Wu, Chao Ma

**Affiliations:** Department of Rehabilitation Medicine, Sun Yat-sen Memorial Hospital, Sun Yat-sen University, Guangzhou, China

**Keywords:** cerebellar fastigial, electrical stimulation, cortical activation, oxygenated hemoglobin concentration, functional near infrared spectroscopy

## Abstract

**Background:**

Electrical stimulation of the cerebellar fastigial nucleus (FNS) has been shown to protect animals against cerebral ischemic injury. However, the changes in cortical activation as a response to FNS have not been illustrated in humans.

**Objective:**

This study aims to detect functional connectivity changes in the brain of stroke patients, and investigate the cortical activation caused by FNS through measuring the oxygenated hemoglobin concentration (HBO) in the cerebral cortex of stroke patients and healthy controls (HCs).

**Methods:**

This study recruited 20 patients with stroke and 20 HCs with all the following factors matched: age, gender and BMI. The experiment session was made up of the pre-task baseline, FNS task period, and post-task baseline. FNS task period contains 5 blocks, each block encompassing the resting state (30 s) and the FNS state (30 s). HBO signals were acquired by functional near-infrared spectroscopy (fNIRS) from the Prefrontal Cortex (PFC), the Motor Cortex (MC) and the Occipital Cortex (OC) throughout the experiment. The Pearson correlation coefficient was used to calculate the resting-state functional connectivity strength between the two groups, and the general linear model (GLM) was used to calculate the activation of 39 fNIRS channels during FNS in stroke patients and HCs, respectively.

**Results:**

The coupling strength of stroke patients were significantly decreased in the following regions: right MC and left MC (*t* = 4.65, *p* = 0.0007), right MC and left OC (*t* = 2.93, *p* = 0.04), left MC and left OC (*t* = 2.81, *p* = 0.04). In stroke patients, the changes in cerebral oxygenated hemoglobin (ΔHBO) among 12 channels (CH) in the bilateral PFC and bilateral MC regions were significantly increased during the FNS state (FDR corrected *p* < 0.05) compared with the resting state. In HCs, only 1 channel was increased (FDR corrected *p* < 0.05) in the left PFC during FNS.

**Conclusion:**

By using the FNS and fNIRS techniques, the characteristics of functional connectivity were found to decrease in stroke patients. It was also noticed that FNS activates the PFC and MC regions. These findings may help to guide functional rehabilitation in stroke patients.

## Introduction

Four pairs of nuclei (fastigial nuclei, emboliform nuclei, globose nuclei and the dentate nuclei) exist in the cerebellum. The cerebellar fastigial nucleus (FNS) is located at the top of the fourth ventricle. According to one MRI study, the FN is approximately 3 × 3 × 3 mm in size (in width, height and length, respectively) (Dimitrova et al., 2002). The FN is well known for its essential role in motor control and the non-motor system (Al-Afif et al., 2019; Zhang X. Y. et al., 2016). It carries out extensive projections to many motor structures, which control the body’s movement by projecting to the reticular structure of the medulla oblongata/brainstem and the primary motor cortex (MC). Eye movement, for example, is governed by the projection of the cranial nucleus in the brainstem. The FN also regulates the cardiovascular system by sending projection fibers to the solitary tract and the paramedian reticular nucleus. In the 1990s, a study discovered a fastigial pressor response (FPR) in cats, whereby the arterial pressure was observed to increase upon stimulation of the FN (Miura and Reis, 1969).

Electrical stimulation of the cerebellar FNS has been proven to improve brain cortical blood perfusion effectively (Golanov and Reis, 1996). It is also effective against cerebral ischemia. Cerebral ischemia can evoke an inflammatory response and secondary brain injury. Researchers found that FNS contributes to decreased infarction volumes, elicits suppression of periinfarction depolarizing waves, promoting axonal regeneration, and inhibiting inflammatory response (Golanov and Reis, 1999; Wang et al., 2019; Xia et al., 2019). Xia et al. (2019) observed that FNS treatment could affect the expression of some inflammatory factors such as caspase-1 and interleukin 1β, besides inhibiting cell apoptosis and promoting neuronal repair and regeneration (Wang et al., 2019). Several basic animal experiments have proven that FNS is involved in regulating activity in other brain regions and modulate vascular activity (Zhang C. et al., 2016; Golanov et al., 2017; Gao et al., 2018). However, clinical applications of this treatment are relatively few, and relevant clinical treatment evidence are still lacking.

Functional imaging technology can be applied to detect brain metabolism, cerebral blood flow (CBF) and other indicators to determine the level of cerebral cortex activity, thus providing a clear image of a person’s state of brain function. Traditional functional imaging methods include functional magnetic resonance imaging (fMRI) (Buxton, 2013) and positron emission tomography (PET) (Hooker and Carson, 2019). Nevertheless, these techniques cannot provide dynamic observation of brain function changes during electrical stimulation. Recently, functional near-infrared spectroscopy (fNIRS) has been widely used in the evaluation of brain function because of its portability, repeatability, and for being non-radiation. fNIRS is a hemodynamics-based neuroimaging technique that provides a non-invasive method for the detection of relative changes in cerebral oxygenated hemoglobin (ΔHBO) at the cortical surface (Lloyd-Fox et al., 2010; Obrig, 2014; Scholkmann et al., 2014). This method is commonly used to detect changes in cerebral hemodynamics during electrical stimulation and rehabilitation tasks in stroke, depression, Parkinson’s and various mental diseases (Noda et al., 2012; Wang et al., 2020; Conceição et al., 2021; Zhang et al., 2021). Kozel et al. (2009) and Li et al. (2019) acquired fNIRS when their subjects were receiving transcranial magnetic stimulation, and they found decreased levels of activation and functional connectivity within the cerebrum. In addition, the simultaneous application of transcranial electrical stimulation or peripheral nerve electrical stimulation combined with fNIRS to explore the mechanism of CBF is an essential innovation in therapeutic interventions (Di Rosa et al., 2019; Huo et al., 2019). The integration of fNIRS and transcranial electrical stimulation therapy also brings great potential for neuroscience research and clinical application of the brain related diseases. All these advantages make fNIRS a suitable technique for detecting activity changes in the brain cortex during FNS.

FNS is a non-invasive brain stimulation technique that transmits bionic low-frequency biological current to the cerebellar FNS through a bilateral mastoid process to modulate CBF and brain function (Hu et al., 2017). This study aims to detect brain functional connectivity changes in stroke patients, and investigate the cortical activation caused by FNS through measuring the concentration of HBO in the cerebral cortex of stroke patients and healthy controls (HCs), respectively.

## Materials and methods

This study was conducted at the Rehabilitation Medicine Department of Sun Yat-sen Memorial Hospital. This study was performed adhering to the Declaration of Helsinki on biomedical research involving stroke patients and healthy subjects. The research protocol was approved by the Sun Yat-sen Memorial Hospital Ethics Committee (SYSEC-KY-KS-2021-251). All participants provided written informed consent before their inclusion in this study. The comprehensive testing lasted for 10 min, including resting and stimulation.

### Participants

Forty right-handed participants comprised of 20 patients with stroke (3 females, 17 males) and 20 sex, age-matched HCs with no history of mental or neurological disorders were recruited from the local community to participate in this fNIRS study. The patients with stroke were recruited from the Department of Rehabilitation Medicine, Sun Yat-sen Memorial Hospital, Guangzhou, China, while the HCs were enrolled from the society. For the patients with stroke, the inclusion criteria for stroke group were as follows: (1) Right-handed; (2) an ischemic stroke as confirmed by imaging (the magnetic resonance imaging or computed tomography) outcomes; (3) unilateral lesions; and (4) more than 4 weeks after the onset of stroke. Meanwhile, the exclusion criteria were as follows: (1) unstable medical condition; (2) any neurological disease except stroke; (3) unable to cooperate with the examination due to depression, anxiety, mania, schizophrenia and/or other mental disorders; (4) skin infections, lesions and/or sensitive tingling in the areas of stimulation; and (5) informed consent could not be obtained from patients or their family members.

The Modified Rankin Scale (MRS), National Institutes of Health Stroke Scale (NIHSS), and Modified Barthel index (MBI) were evaluated by trained attending physicians to assess patients’s neurological and motor functional recovery. The Body mass index (BMI) and blood pressure were extracted from the electronic medical records of patients in hospital.

### Study design

**FIGURE 1:**
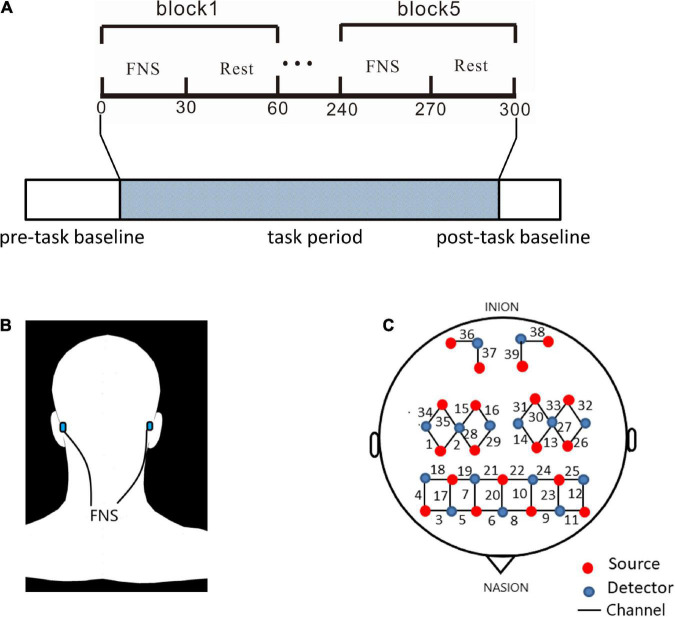
Experiment equipment and procedures. **(A)** The experimental process consisted pre-task baseline, task period, and post-task baseline. The block design in task period is made up of 5 cycles of 2 states: 30 s of FNS and 30 s of rest. **(B)** Location of electrodes for FNS: bionic low-frequency biological current from 2 transcutaneous electrodes is transmitted along the mastoid process to the fastigial nucleus. **(C)** The source optodes, detector optodes and channels: 39 Channels according to the 10/10 system. 6 cerebral areas were measured: LPFC, RPFC, LMC, RMC, LOC, and ROC.

The experimental section consisted of a 40-s pre-task baseline, a 300-s FNS task period, and a 20-s post-task baseline (Figure 1A). The 300-s FNS task period was made up of 5 block sets, with each block containing an FNS condition (30 s) and a resting condition (30 s). Participants were asked to sit in a quiet, dark environment to avoid noise and light interference, and the order of tasks was not revealed before the experiment. The fNIRS measurements were taken through the whole experimental process. During the FNS condition, electrodes of the FNS electrical stimulator (CVFT-201, QianKang electrical stimulator, ShangHai, China) were attached to the bilateral mastoid (Figure 1B). FNS was performed using model 3 (136 Hz, strength: 45–90%) according to the manufacturer’s instructions.

### Functional near-infrared spectroscopy

A continuous-wave, multi-channel fNIRS system (NirSmart, Danyang Huichuang Medical Equipment Co., Ltd.) was used to measure HBO and deoxygenated hemoglobin (HBR) with a sampling rate of 11 Hz. The wavelengths of light for this measurement system were between 730 and 850 nm. As shown in Figure 1C, 39 fNIRS channels were positioned over the PFC (LPFC/RPFC), MC (LMC/RMC) and OC (LOC/ROC) regions. The fNIRS measured light intensity signals through 19 light source probes and 15 detector probes. Concentration changes of HBO were calculated by the light intensity obtained from fNIRS.

### Data preprocessing

The data recorded from fNIRS were preprocessed and analyzed using the NirSpark software package, which runs in MATLAB (Mathworks, United States). Data preprocessing included several steps. The raw light intensity was converted to optical density according to the modified Beer-Lambert Law (Kocsis et al., 2006). Remove both baseline shifts and spike artifacts by using parameter-free motion correction method (Hou et al., 2021). The band-pass filtering of the optical density signals was set between 0.01 and 0.2 Hz to remove baseline drift and physiological noise (Zheng et al., 2019; Si et al., 2021). The optical density transformed to HBO and HBR concentrations (Tak and Ye, 2014). Because the HBO has a better signal-to-noise ratio than HBR, we choose the HBO as our primary indicator in the following analysis. The hemodynamic response function (HRF) initial time was set to –5 s while the end time was set to 50 s (with “–5 to 0 s” as the baseline state and “0–50 s” as the state for a block paradigm). With the FNS duration set to 30 s, the HBO of each five-block paradigm were superimposed and averaged to produce an average block result.

### Analysis of functional near-infrared spectroscopy data

For each participant, functional connectivity was calculated by conducting the Pearson correlation analyses between time series of each pair channel. The Pearson correlation was calculated for the pre-task baseline between any two channels to generate a 39 × 39 correlation matrix for each participant. Furthermore, the 39 channels were divided into six regions of interest (ROI), including LPFC, RPFC, LMC, RMC, LOC, and ROC. We calculated the functional connection matrix between the six ROI in the same way. For any two ROI, the mean value of the functional connection value of all channel pairs between them is used as the functional connection value between the two ROI.

The mean time course for one subject is expressed as X = [xi(t)t = 1,2,3,….N], where xi(t)t = 1,2,3,….N is the mean time series of the region. The formula for calculation is as follows: $\bar{\text{x}}$

xi = the average of xi


(1) $$r\,\left(x_{i},x_{j}\right)=\frac{\sum_{t=1}^{N}\left[x_{i}\,\left(t\right)-{\bar{x}}_{i}\right]\,\left[x_{j}\,\left(t\right)-{\bar{x}}_{j}\right]}{\sqrt{\sum_{t=1}^{N}\left[x_{i}\left(t\right)-{\bar{x}}_{i}\right]{}^{2}}\,\sqrt{\sum_{t=1}^{N}\left[\left[x_{j}\left(t\right)-{\bar{x}}_{j}\right]\right]{}^{2}}}$$

According to the waveforms of individuals in all 39 channels, each channel’ s averaged waveforms of HBO and HBR changes of all participants in the stroke groups were obtained. The general linear model (GLM) (Uga et al., 2014) was used to analyze the preprocessed HBO data of each channel for each subject to identify cerebral areas that were significantly activated by FNS. GLM establishes an ideal HRF for each subject task, allowing the calculation of beta values that reflect the level of cerebral cortex activation according to the matching degree of actual HRF and excellent HRF values. The results of this study are based on beta values.

### Statistical analysis

Data normality was tested by the Shapiro-Wilk test. The Mann-Whitney *U*-test was used for age and height difference in stroke and HCs, while gender was tested by the chi-square test. A two-sample *t*-test was used to compare the characteristics such as weight, BMI and BP between the stroke group and the HCs.

In the activation analysis, the beta values of the stroke group and the HCs were analyzed with a single sample *t*-test and two-sample *t*-test on each channel by using the NirSpark software. Statistical results were corrected by FDR correction (*p* < 0.05).

To find the difference in functional connectivity between the HCs and the stroke group, two-sample *t*-tests were performed between groups to identify correlation in each ROI, and the results were corrected by FDR multiple hypothesis tests.

## Results

### Demographic and characteristics

The demographic characteristics comparisons of the two groups are listed in Table 1. The stroke group included 17 males and 3 females. The HCs included 16 males and 4 females. Participants with stroke and HCs showed no difference in age (*z* = 1.93, *p* > 0.05), gender (chi-square = 0.17, *p* > 0.05), BMI (*t* = 1.18, *p* > 0.05), or blood pressure (*t* = 0.73, *p* > 0.05; *t* = –0.55, *p* > 0.05). Among them, 11 patients had unilateral basal ganglia infarction, 5 patients had brainstem infarction (Supplementary Table 1). The mean time after stroke of stroke group was 4 months (*SD* = 2.1), the mean MRS, NIHSS and MBI scores of patients were 3 (*SD* = 0.7), 10 (*SD* = 4.5) and 45 (*SD* = 12), respectively.

**TABLE 1:** Characteristics comparison between stroke group and HCs.

	Stroke (*n* = 20)	HCs (*n* = 20)	T/Z/χ^2^	*p*
Age (years)	42 (30.0, 49.75)	30 (28.39)	1.93	0.056
Gender (M/F)	17/3	16/4	0.17	0.677
Weight (kg)	63 (8.9)	58 (8.4)	1.77	0.085
Height (cm)	173 (162.5, 175.8)	165.5 (160.3, 172.3)	1.66	0.096
BMI (kg/m^2^)	22 (2.3)	21 (1.8)	1.18	0.242
Systolic blood pressure (mmHg)	126 (17.9)	123 (9.6)	0.73	0.473
Diastolic blood pressure (mmHg)	82 (13.2)	84 (6.1)	–0.55	0.584
Time poststroke (month)	4 (2.1)	–	–	–
MRS	3 (0.7)	–	–	–
NIHSS	10 (4.5)	–	–	–
MBI	45 (12)	–	–	–

HCs, Healthy controls; BMI, Body mass index; MRS, Modified Rankin Scale; NIHSS, National Institutes of Health Stroke Scale; MBI, Modified Barthel index.

### Decreased functional connectivity in stroke patients

**FIGURE 2:**
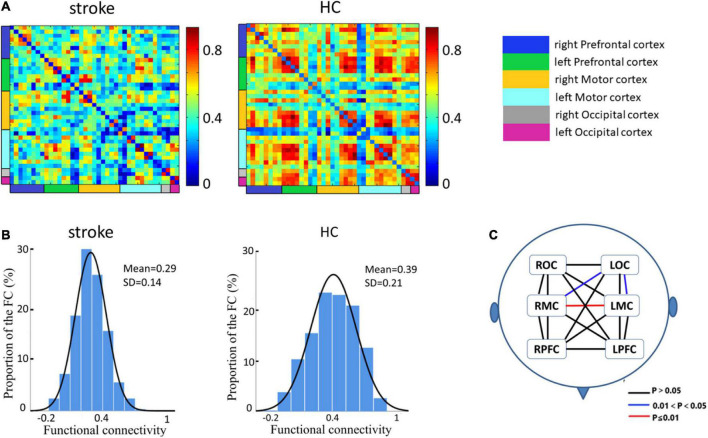
Spatial patterns of the functional connectivity in the FNS state and resting state. **(A)** Networks of FNS-related changes in functional connectivity between stroke patients and HCs. **(B)** Histograms of functional connectivity distribution in the two groups. **(C)** Functional connectivity in patients with stroke showed significant decreases in different cerebral regions when compared with HCs.

Figure 2A employs a 39 × 39 matrix figure to show averaged connectivity strength during FNS by group. Data showed that the average connectivity strength in stroke patients was much lower [e.g., in the MC and occipital cortex (OC)] than that in the HCs. The mean values and standard deviations of connectivity strength were 0.29 ± 0.14 for stroke patients, and 0.39 ± 0.21 for HCs (Figure 2B). Figure 2C shows the difference in functional connection strength between the two groups in six ROI. Compared with the HCs, stroke patients showed decreased functional connectivity between RMC and LMC (*t* = 4.65, *p* = 0.0007), RMC and LOC (*t* = 2.93, *p* = 0.04), and LMC and LOC (*t* = 2.81, *p* = 0.04). The results were corrected by FDR.

### Analysis of cortical activation

**FIGURE 3:**
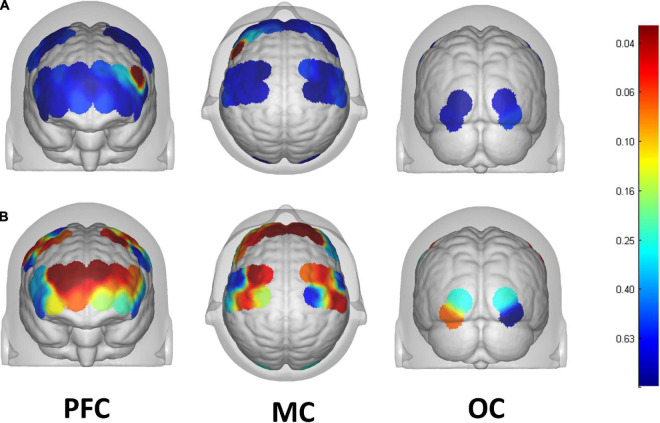
Brain map of activation regions during FNS by using beta values of GLM analysis in the **(A)** HCs, and the **(B)** stroke patients. The color bar represents the *P*-value of beta values.

**FIGURE 4:**
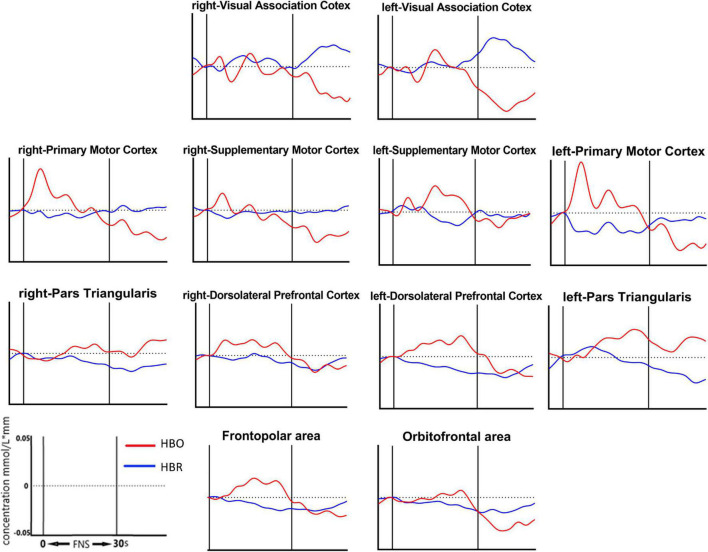
Grand average waveforms of HBO changes during FNS in each ROI based on Brodmann in stroke patients. The vertical axis represents concentration changes of HBO and HBR; while the horizontal axis represents the latency. The start time of the FNS period was defined as 0 s. The ROI contained 12 cerebral areas, which include the bilateral Visual Association Cortex, bilateral Primary Motor Cortex, bilateral Supplementary Motor Cortex, bilateral Pars Triangular, bilateral Dorsolateral Prefrontal Cortex, Frontopolar area and Obitorfrontal area.

13 of the 39 channels were significantly activated in the 2 groups distributed across the 12 Brodmann regions (Table 2) where distributed according MNI coordination (Supplementary Table 3). In stroke patients, the ΔHBO among several channels in the bilateral PFC (CH7, CH19, CH20, CH21, CH22, CH24) and bilateral MC (CH13, CH14, CH28, CH30, CH34, CH35) regions were significantly increased during the FNS state (*p* < 0.05 FDR correction, the effect size of all channels can be found in Supplementary Table 2) when compared with the resting state. In HCs, the ΔHBO in CH25 of the left Pars Triangular was significantly increased as compared to the resting state. Figure 3 shows brain maps with activation regions during FNS being highlighted by using beta values of GLM analysis in (A) HCs and (B) stroke patients. Figure 4 shows dynamic concentration changes in the average HBO of stroke patients at different Brodmann regions over time (–5 to 60 s, 0–30 s represent the FNS period). The concentration of HBO gradually increased with the initiation of FNS. It reached the peak value after 10 s and then dropped rapidly in the bilateral MC.

**TABLE 2:** Channels with HBO significantly increased when compared with the resting baseline during FNS.

ROI of cortical area	Channels
L-DLPC	9 23 24*
R-DLPC	3 17 19* 28*
L-PT	11 12 25^#^
R-PT	3 4 18
L-PMC	31 30* 32 33
R-PMC	15 16 34* 35*
L-SMC	13* 14* 26 27
R-SMC	1 2 28* 29
L-VAC	38 39
R-VAC	36 37
Obitorfrontal area	5 6 8
Frontopolar area	7* 9 10 20* 21* 22*

L, left; R, right; DLPC, Dorsolateral Prefrontal Cortex; PT, Pars Triangular; PMC, Primary Motor Cortex; SMC, Supplementary Motor Cortex; VAC, Visual Association Cortex.

*p < 0.05 in the stroke group.

^#^p < 0.05 in the stroke group and HCs.

## Discussion

In this study, the fNIRS approach was used to study cortex functional connectivity and activation characteristics in the stroke patient and HCs. From the functional connectivity perspective, the correlation coefficients between bilateral MC and left OC were found to decrease in stroke patients compared with the HCs during the resting state; from the cortex activation perspective, significant activation was observed in the PFC and MC of stroke patients during FNS. Moreover, FN was also found to activate part of the LPFC in the HCs.

Based on the results of this study, the functional connectivity strength between RMC and LMC, RMC and LOC, and LMC and LOC were significantly decreased in stroke patients compared with participants in the HCs. And the strength of functional connection in both hemispheres decreased asymmetrically. Neural plasticity is an important factor that reflects cortical reorganization after stroke, and functional connection is one of the main indicators of neural plasticity. The MC is the core of the brain that plans and executes volitional movements (Svoboda and Li, 2018), whereas the OC is associated with peripheral sensory information (Song et al., 2017). The results of this study are consistent with previous studies that reported a high correlation between decreased sensorimotor connectivity and clinical motor functional defect assessment (Volz et al., 2016). Studies found that focal cerebral ischemic injury can lead to a decline in the complete network of sensorimotor connections, especially in the early stage of stroke (Park et al., 2011). It can also affect functional connections between the hemispheres of the undamaged lateral cortex (Green et al., 2018). Although all patients in the stroke patient group of this study suffered from long-term unilateral ischemic brain injury and most of the infarcts are located in the left basal ganglia, with only a few patients that had large frontal parietal and temporal lobe infarction on left hemisphere, results showed weakened intra-hemispheric and inter-hemispheric brain functional connectivity. The results of our study suggest that our stroke patients still need rehabilitation therapy to improve the motor function of the affected limbs and improve the level of motor functional connection between the hemispheres.

Results also showed that the HBO of the bilateral PFC and bilateral MC significantly increased during the FNS period in stroke patients and HCs. This outcome indicates that FNS can activate parts of the brain in the PFC and MC. HBO in the MC increased with FNS but gradually returned to baseline when FNS was stopped (Figure 4). These results are in line with those of previous studies (Steriade, 1995; Golanov and Reis, 1996; Watson et al., 2009, 2014), where EEG examination showed that the brain-electric activity began to increase after FNS. CBF was also found to increase in 4 s, peaked around 8 s, and then gradually dropped to the baseline level. A plausible explanation to these results may be the ability of FNS in increasing CBF by evoking vasodilation neurons. The HBO signal could reflect the dynamic changes in the neural and functional activity of the cerebral cortex due to the neurovascular coupling mechanism (Claassen et al., 2021). Besides the influence of CBF, FNS also activates the intrinsic neurons in the cerebral cortex through neural pathways (Reis et al., 1997). Fibers from the FNS cross to the opposite side of the cerebellar vermis to form an uncinate cerebellar bundle (Haroian et al., 1981). This nerve bundle projects to the dorsal tegmental area of the midbrain, continues to rise into the thalamus, and finally terminates in the ventromedial nucleus of the thalamus dorsal medial nucleus. Due to the extensive relationship between the dorsal medial thalamic nucleus and the prefrontal cortex (PFC), the FNS could activate the PFC (Steriade, 1995). The present results are significant as the activation of PFC and MC by FNS provides a certain therapeutic basis and plays a guiding role in the rehabilitation of stroke patients.

## Conclusion

This study illustrated that FNS induced activation in the brain regions of PFC and MC, with the FC values between bilateral MC and left OC being significantly decreased in stroke patients. The results of this study provide evidence for the clinical application and curative effect guidance of FNS treatment in the rehabilitation of patients with stroke and brain injury.

## Limitations

Due to the limitation of the fNIRS technology, it was not possible to measure the subcortical structures and cerebellum. In the future, the mechanisms related to the neural conduction pathways between cerebellar FN and cortex should be further explored to provide more accurate evidence that would be critical for potential clinical application. Besides, the sample size in this study was not large enough as well.

## Data availability statement

The raw data supporting the conclusions of this article will be made available by the authors, without undue reservation.

## Ethics statement

The studies involving human participants were reviewed and approved by the Sun Yat-sen Memorial Hospital Ethics Committee. The patients/participants provided their written informed consent to participate in this study.

## Author contributions

HM and YZ performed the experiment and wrote the manuscript. ZX collected the data. SF and XW contributed significantly to the data analysis. JX contributed to data processing. CM and SW contributed equally to design the experiment. All authors read and approved the final version of the manuscript.
