# Supplementary material for: Increased cerebral cortex activation in stroke patients during electrical stimulation of cerebellar fastigial nucleus with functional near-infrared spectroscopy
Source: Front Neurosci. 2022 Aug 18;16:895237. doi: 10.3389/fnins.2022.895237 (PMC9433974; doi:10.3389/fnins.2022.895237)
Supplement: Supplementary file 1 [file Data_Sheet_1.docx]

Supplementary Material

# Supplementary Tables

Table S1 Stroke Patients Characteristics

| Patients | Age | Gender | Time poststroke  (months) | Affected Hemiplegia | Site of lesion | NIHSS | MBI | MRS |
| --- | --- | --- | --- | --- | --- | --- | --- | --- |
| 1 | 57 | M | 2.0 | R | LBG | 3 | 50 | 3 |
| 2 | 57 | M | 3.0 | R | LBG, LCR, L brainstem | 10 | 35 | 4 |
| 3 | 30 | F | 5.0 | R | LBG, LCR, LFL | 14 | 15 | 5 |
| 4 | 50 | M | 4.5 | R | LBG | 15 | 40 | 3 |
| 5 | 18 | M | 1.5 | R | LT, brainstem | 12 | 30 | 4 |
| 6 | 42 | M | 2.0 | R | LBG | 16 | 45 | 3 |
| 7 | 47 | M | 3.0 | R | LBG, LCR | 10 | 40 | 4 |
| 8 | 28 | F | 1.5 | R | LFL, LPL, LTL | 8 | 60 | 3 |
| 9 | 33 | M | 3.0 | R | LBG | 14 | 45 | 4 |
| 10 | 52 | M | 5.0 | R | LBG, LT | 8 | 35 | 4 |
| 11 | 53 | M | 5.0 | R | LFL, LPL, LTL,LCR,LBG | 12 | 60 | 3 |
| 12 | 39 | M | 8.5 | R | LFL | 5 | 45 | 3 |
| 13 | 47 | M | 8.0 | R | L brainstem | 13 | 45 | 3 |
| 14 | 49 | M | 5.5 | R | LFL, LPL, LTL, LBG | 5 | 55 | 3 |
| 15 | 28 | F | 6.0 | R | LFL, LTL | 7 | 50 | 2 |
| 16 | 42 | M | 7.0 | R | LPL, LOL, brainstem | 4 | 65 | 2 |
| 17 | 45 | M | 4.0 | R | LCC | 16 | 55 | 3 |
| 18 | 33 | M | 4.0 | R | brainstem | 18 | 30 | 4 |
| 19 | 27 | M | 2.5 | R | LPL,LFL,LOL | 11 | 50 | 3 |
| 20 | 35 | M | 1.5 | R | LPL,LFL, LTL,LBG | 4 | 55 | 3 |

Abbrevitions: BG, basal ganglia; CC, corpus callosum; CR, corona, radiate; F, female; FL, frontal lobe; L, left; M, male; MBI, modified barthel index; MRS, modified Rankin Scale; NIHSS, National Institutes of Health Stroke Scale; OL, occipital lobe; PL, parietal lobe ;R, right; T, thalamus; TL, temporal lobe.

Table S2. The effect size about beta value analyse of 39 channels in stroke patients.

| Channel | T | df | p | Effect size  (Cohen’s d) |
| --- | --- | --- | --- | --- |
| Ch1 | -0.65838 | 19 | 0.56 | -0.3 |
| Ch2 | 2.2519 | 19 | 0.07 | 1.0 |
| Ch3 | -0.82858 | 19 | 0.48 | -0.38 |
| Ch4 | -1.228 | 19 | 0.31 | -0.56 |
| Ch5 | 1.998 | 19 | 0.11 | 0.91 |
| Ch6 | 2.4967 | 19 | 0.06 | 1.14 |
| Ch7 | 3.3399 | 19 | 0.02 | 1.53 |
| Ch8 | 1.7868 | 19 | 0.14 | 0.81 |
| Ch9 | 1.5971 | 19 | 0.19 | 0.73 |
| Ch10 | 2.3292 | 19 | 0.06 | 1.06 |
| Ch11 | 0.82447 | 19 | 0.48 | 0.37 |
| Ch12 | -1.198 | 19 | 0.31 | -0.55 |
| Ch13 | 3.8612 | 19 | 0.01 | 1.77 |
| Ch14 | 2.7188 | 19 | 0.04 | 1.24 |
| Ch15 | 1.9316 | 19 | 0.12 | 0.88 |
| Ch16 | 0.56519 | 19 | 0.60 | 0.25 |
| Ch17 | 1.0577 | 19 | 0.36 | 0.48 |
| Ch18 | 1.1784 | 19 | 0.31 | 0.54 |
| Ch19 | 3.7528 | 19 | 0.01 | 1.72 |
| Ch20 | 2.7808 | 19 | 0.04 | 1.27 |
| Ch21 | 3.7932 | 19 | 0.01 | 1.74 |
| Ch22 | 3.6897 | 19 | 0.01 | 1.69 |
| Ch23 | 2.4127 | 19 | 0.06 | 1.10 |
| Ch24 | 2.9619 | 19 | 0.03 | 1.36 |
| Ch25 | 2.3583 | 19 | 0.06 | 1.08 |
| Ch26 | 0.78594 | 19 | 0.49 | 0.36 |
| Ch27 | 0.27056 | 19 | 0.81 | 0.12 |
| Ch28 | 2.9876 | 19 | 0.03 | 1.37 |
| Ch29 | 2.3952 | 19 | 0.06 | 1.09 |
| Ch30 | 2.7229 | 19 | 0.04 | 1.24 |
| Ch31 | 1.8269 | 19 | 0.14 | 0.83 |
| Ch32 | 1.3153 | 19 | 0.28 | 0.60 |
| Ch33 | 2.5459 | 19 | 0.059 | 1.16 |
| Ch34 | 2.8049 | 19 | 0.04 | 1.28 |
| Ch35 | 3.1982 | 19 | 0.03 | 1.46 |
| Ch36 | 0.16237 | 19 | 0.87 | 0.07 |
| Ch37 | 1.4831 | 19 | 0.22 | 0.68 |
| Ch38 | 2.3306 | 19 | 0.06 | 1.06 |
| Ch39 | 1.4742 | 19 | 0.22 | 0.67 |

The effect size will be submitted as supplementary materials.

Table S3. The MNI coordinates corresponding to the 39 channels

| Channel | X | Y | Z | Brodmann area |
| --- | --- | --- | --- | --- |
| Ch1 | 61 | 1 | 44 | BA6 |
| Ch2 | 52 | 4 | 53 | BA6 |
| Ch3 | 53 | 46 | -7 | BA46 |
| Ch4 | 60 | 31 | 6 | BA45 |
| Ch5 | 38 | 65 | -3 | BA11 |
| Ch6 | 11 | 74 | 1 | BA11 |
| Ch7 | 25 | 70 | 15 | BA10 |
| Ch8 | -17 | 73 | 1 | BA11 |
| Ch9 | -41 | 60 | -3 | BA10 |
| Ch10 | -28 | 66 | 15 | BA10 |
| Ch11 | -53 | 40 | -8 | BA45 |
| Ch12 | -58 | 24 | 7 | BA45 |
| Ch13 | -40 | 8 | 61 | BA6 |
| Ch14 | -28 | 8 | 68 | BA6 |
| Ch15 | 46 | -18 | 66 | BA4 |
| Ch16 | 35 | -16 | 73 | BA6 |
| Ch17 | 47 | 55 | 10 | BA46 |
| Ch18 | 53 | 40 | 23 | BA45 |
| Ch19 | 35 | 57 | 26 | BA46 |
| Ch20 | -2 | 69 | 16 | BA10 |
| Ch21 | 14 | 67 | 29 | BA10 |
| Ch22 | -12 | 65 | 29 | BA10 |
| Ch23 | 47 | 50 | 9 | BA46 |
| Ch24 | -36 | 56 | 26 | BA46 |
| Ch25 | -52 | 36 | 23 | BA45 |
| Ch26 | -60 | 0 | 41 | BA6 |
| Ch27 | -50 | 4 | 54 | BA6 |
| Ch28 | 43 | 9 | 60 | BA6 |
| Ch29 | 30 | 10 | 66 | BA6 |
| Ch30 | -43 | -19 | 67 | BA4 |
| Ch31 | -30 | -17 | 74 | BA6 |
| Ch32 | -63 | -24 | 46 | BA4,BA1 |
| Ch33 | -54 | -21 | 59 | BA4,BA3 |
| Ch34 | 64 | -23 | 49 | BA4,BA1 |
| Ch35 | 56 | -18 | 58 | BA4,BA1 |
| Ch36 | 33 | -97 | -14 | BA18 |
| Ch37 | 27 | -103 | 2 | BA17 |
| Ch38 | -32 | -98 | -15 | BA18 |
| Ch39 | -23 | -106 | 1 | BA17 |

The MNI coordinates will be submitted as supplementary materials.
